# Dataset of RNA-seq profiles from THP-1-derived macrophages pretreated with AZD7648 and stimulated with CT DNA

**DOI:** 10.1016/j.dib.2026.113124

**Published:** 2026-08-03

**Authors:** Qiang Li, Pei-Wen Zhan, Xian Hong, Yong-Pei Li, Xin-Yue Pei, Xiao-Hui Du, Ming Sui, Tao Wang

**Affiliations:** a Laboratory of Protein Structure and Function, Institute of Medicine and Pharmacy, Qiqihar Medical University, Qiqihar, Heilongjiang, 161006, China; b Department of Medical Technology, Qiqihar Medical University, Qiqihar, Heilongjiang, 161006, China; c Department of Basic Medical Science, Qiqihar Medical University, Qiqihar, Heilongjiang, 161006, China; d College of Pharmacy, Qiqihar Medical University, Qiqihar, Heilongjiang, 161006, China

**Keywords:** RNA-seq, Transcriptomics, DNA-PKcs, AZD7648, Innate immunity

## Abstract

This article describes an RNA-seq dataset generated from THP-1-derived macrophages under four treatment conditions: untreated control, DNA-dependent protein kinase catalytic subunit (DNA-PKcs) inhibitor AZD7648 alone, calf thymus DNA (CT DNA) alone, and AZD7648 pretreatment followed by CT DNA stimulation. Each condition included three biological replicates, yielding 12 transcriptome samples in total. Libraries were sequenced on the Illumina NovaSeq 6000 platform using paired-end 150-bp reads. The dataset includes raw sequencing reads, gene-level expression matrices, and associated quality control metrics. All RNA samples exhibited RIN values between 9.5 and 9.9. Clean reads ranged from 54.83 to 70.25 million per sample, with Q30 values of 97.18%-97.34%, total mapping rates of 98.24%-98.51%, and unique mapping rates of 95.55%-95.81%. This dataset provides a resource for comparative transcriptomic analyses of cytosolic DNA stimulation and DNA-PKcs inhibition in macrophage cells and may support reuse in studies of innate immune signaling, cytosolic DNA sensing, and pathway-focused reanalysis.

Specifications Table

**Table:** 

Subject	Biology
Specific subject area	Innate immune response; THP-1 macrophage transcriptomics; RNA-Seq dataset
Type of data	Table, Figure, Raw, Analysed, Processed.
Data collection	THP-1-derived macrophages were divided into four groups: untreated control, AZD7648 alone, CT DNA alone, and AZD7648 + CT DNA. Total RNA was extracted, and libraries were prepared with the VAHTS mRNA-seq V2 Library Prep Kit and sequenced on the Illumina NovaSeq 6000 platform. Raw reads were quality-filtered, aligned to the human reference genome, and gene expression was quantified.
Data source location	Qiqihar Medical University, 333 Bukui North Street, Qiqihar, China
Data accessibility	Repository name: NCBI Sequence Read Archive (SRA) Data. Data identification number: PRJNA1449245, with BioSamples including SAMN57115764-SAMN57115775. Direct URL to data: https://www.ncbi.nlm.nih.gov/bioproject/PRJNA1449245
Related research article	None

## 1 Value of the Data

• This dataset provides RNA-seq profiles from THP-1-derived macrophages exposed to AZD7648, CT DNA, and their combination under a four-group experimental design with biological replicates.
• The dataset can be used to investigate the role of DNA-PKcs in cytosolic DNA sensing and innate immune responses in macrophages.
• The dataset can be used for comparative analysis of transcriptional responses associated with cytosolic DNA stimulation and DNA-PKcs inhibition.
• The dataset can be reused cross-disciplinarily for research on cGAS–STING modulators and STING-based cancer immunotherapy, matching the translational STING therapeutic focus in Background.

## 2 Background

The cyclic GMP-AMP synthase (cGAS)-stimulator of interferon genes (STING) pathway is the primary innate immune sensor of cytosolic DNA, driving type I interferon and inflammatory cytokine production essential for host defense [1,2]. Dysregulation of this pathway contributes to autoimmune diseases, while its activation in tumors enhances anti-tumor immunity [3,4], making STING agonists/inhibitors promising for cancer immunotherapy and autoinflammatory conditions [[5], [6], [7], [8]].

Beyond its canonical role in DNA repair, DNA-dependent protein kinase catalytic subunit (DNA-PKcs) has emerged as a complex regulator of cytosolic DNA sensing. DNA-PKcs can activate IRF3 and type I interferons independently of cGAS-STING [9]. However, other studies report that it promotes or suppresses cGAS activity by phosphorylating distinct residues of cGAS [[10], [11], [12]]. Additionally, DNA-PKcs has been shown to bind cyclic dinucleotides and thereby restrain STING activation [13]. These conflicting observations indicate that DNA-PKcs functions as a bidirectional modulator of innate immunity, highlighting the need for transcriptome-level datasets to define its effects. To address this, we generated RNA-seq data from THP-1-derived macrophages treated with the selective DNA-PKcs inhibitor AZD7648, Calf thymus DNA (CT DNA), or their combination. This dataset provides a resource for investigating whether and how DNA-PKcs inhibition modulates STING-dependent gene expression and broader innate immune programs.

## 3 Data Description

The dataset is deposited in the NCBI Sequence Read Archive (SRA) under BioProject accession PRJNA1449245 and consists of raw Illumina RNA-seq data in fastq.gz format. The repository is organized as follows (Table 1). A detailed description of the experimental conditions for each BioSample identifier was listed in Table 2.

**Table 1:** Organization of the repository of the RNA-Seq dataset.

Data level	Content	Description
BioProject	PRJNA1449245	Complete project container for the RNA-seq dataset of THP-1-derived macrophages
BioSample	Individual samplesSAMN57115764 - SAMN57115775	Metadata for THP-1-derived macrophages under 4 treatment conditions, including 3 biological replicates per group

**Table 2:** Experimental design and sample IDs deposited in the NCBI database.

Group	Sample	Treatment condition	Biological replicates	NCBI Bioproject ID	NCBI Biosample IDs
CON	CK	Untreated control	3	PRJNA1449245	SAMN57115764, SAMN57115765, SAMN57115766
AZD	Treat1	AZD7648 alone	3	PRJNA1449245	SAMN57115767, SAMN57115768, SAMN57115769
DNA	Treat2	CT DNA alone	3	PRJNA1449245	SAMN57115770, SAMN57115771, SAMN57115772
AZD+DNA	Treat3	AZD7648 + CT DNA	3	PRJNA1449245	SAMN57115773, SAMN57115774, SAMN57115775

Abbreviations: CON, untreated control; AZD, AZD7648 treatment; DNA, CT DNA stimulation; DNA+AZD, AZD7648 pretreatment combined with CT DNA stimulation; CT, calf thymus.

The dataset consists of RNA-seq data from THP-1-derived macrophages assigned to four treatment groups: untreated control (CON), AZD7648 alone (AZD), CT DNA alone (DNA), and AZD7648 pretreatment followed by CT DNA stimulation (AZD + DNA). Each group contained three biological replicates, resulting in 12 samples in total. Sequencing was performed on the Illumina NovaSeq 6000 platform using paired-end 150-bp reads.

The RIN values of all RNA samples were between 9.5 and 9.9, confirming satisfactory RNA integrity. After filtering, the number of clean reads obtained for each sample ranged from 54.83 to 70.25 million, with Q30 values ranging from 97.18% to 97.34%, indicating high sequencing quality (Table 3). Read alignment statistics are summarized in Table 4. The total mapping rates ranged from 98.24% to 98.51%, and the uniquely mapped read rates ranged from 95.55% to 95.81%, demonstrating the high quality of the sequencing data and alignment results.

**Table 3:** Summary of clean sequencing data quality for all samples.

Group	Sample	RIN value	Clean Reads (M)	Clean Bases (Gb)	Avg read length (bp)	Q20 (%)	Q30 (%)	GC (%)
CON	CK-1	9.6	56.33	8.26	147	99.65%	97.22%	51.21%
	CK-2	9.7	59.32	8.67	146	99.67%	97.34%	51.27%
	CK-3	9.7	61.20	9.00	147	99.65%	97.20%	50.60%
AZD	Treat1–1	9.9	66.46	9.77	147	99.66%	97.25%	51.08%
	Treat1–2	9.9	54.83	8.04	147	99.65%	97.24%	50.58%
	Treat1–3	9.7	70.25	10.29	146	99.65%	97.20%	50.98%
DNA	Treat2–1	9.7	60.91	8.91	146	99.65%	97.20%	50.66%
	Treat2–2	9.8	64.22	9.41	147	99.66%	97.30%	49.92%
	Treat2–3	9.8	63.78	9.33	146	99.66%	97.25%	51.13%
AZD+DNA	Treat3–1	9.5	65.17	9.52	146	99.65%	97.18%	51.61%
	Treat3–2	9.8	56.29	8.27	147	99.66%	97.28%	50.42%
	Treat3–3	9.8	66.52	9.71	146	99.66%	97.30%	50.68%

Note. RIN value represents RNA integrity number. Clean Reads and Clean Bases represent post-filtering reads and bases, respectively. Q20 and Q30 indicate the percentages of bases with Phred quality scores of at least 20 and 30.

**Table 4:** Summary of read alignment statistics for all samples.

Group	Sample	Total mapped (%)	Uniquely mapped (%)	Multiple mapped (%)	Properly paired (%)
CON	CK-1	98.47	95.77	2.70	94.53
	CK-2	98.49	95.64	2.85	94.50
	CK-3	98.50	95.81	2.69	94.52
AZD	Treat1–1	98.32	95.66	2.66	94.33
	Treat1–2	98.24	95.55	2.69	94.19
	Treat1–3	98.25	95.56	2.70	94.17
DNA	Treat2–1	98.51	95.81	2.69	94.59
	Treat2–2	98.38	95.71	2.66	94.37
	Treat2–3	98.44	95.69	2.75	94.49
AZD+DNA	Treat3–1	98.51	95.70	2.80	94.51
	Treat3–2	98.32	95.71	2.61	94.39
	Treat3–3	98.46	95.75	2.71	94.56

Note. Percentages are relative to total reads for each sample. Properly paired indicates the proportion of read pairs mapped in proper pairs.

**Fig. 1:**
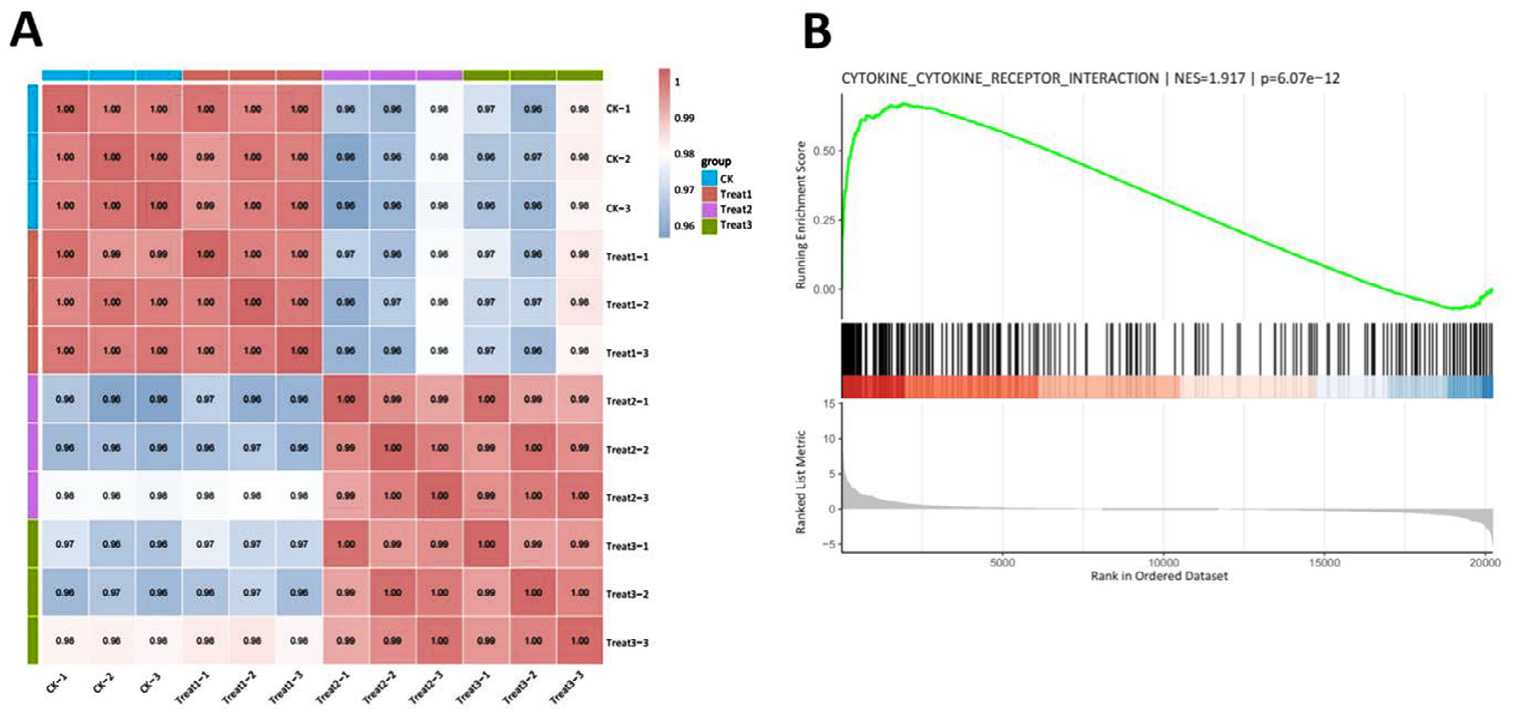
Sample correlation analysis and GSEA of the RNA-seq dataset.(A) Sample correlation heatmap of all RNA-seq samples. The color scale represents the pearson correlation coefficient between samples, ranging from blue (low correlation) to red (high correlation). (B) GSEA plot comparing the CON and DNA groups, showing significant enrichment of the cytosolic DNA‑sensing pathway.

A sample correlation heatmap is shown in Fig. 1A, where the color scale represents the correlation coefficient between samples. The heatmap showed high concordance among biological replicates and distinct transcriptomic separation between CT DNA-transfected samples and controls. In addition, gene set enrichment analysis (GSEA) comparing the CON and DNA groups indicated significant enrichment of the cytosolic DNA-sensing pathway, indicating that CT DNA stimulation effectively activated the expected innate immune pathway in this experimental system (Fig. 1B).

**Fig. 2:**
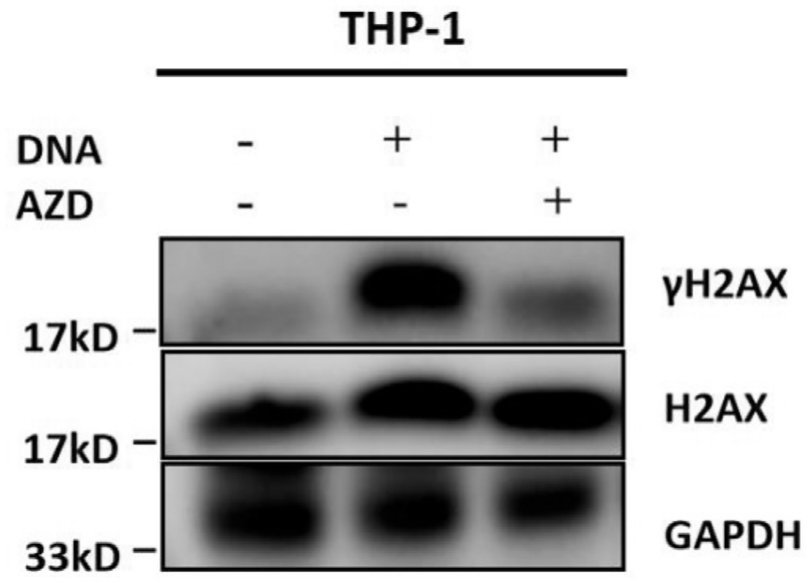
AZD7648 suppresses CT DNA-induced γH2AX accumulation. PMA-differentiated THP1 cells were pretreated with AZD7648 for 1 h and then transfected with CT DNA for 6 h, followed by Western blot analysis of γH2AX.

To further verify the pharmacological activity of AZD7648 in our experimental system, we examined H2AX phosphorylation (γH2AX), which is the substrate of DNA-PKcs in response to transfected DNA [9]. CT DNA transfection markedly increased γH2AX levels compared with the control group, consistent with activation of DNA-PKcs-related signaling following DNA stimulation (Fig. 2). This increase was clearly suppressed by AZD7648 treatment, indicating effective inhibition of DNA-PKcs activity in CT DNA-transfected THP-1-derived macrophages. This validation supports the reliability of the RNA-seq dataset generated from AZD7648-treated samples.

## 4 Experimental Design, Materials and Methods

### 4.1 Cell culture and treatment design

THP-1 cells were purchased from National Infrastructure of Cell Line Resource, and cultured in RPMI-1640 medium supplemented with 10% fetal bovine serum and 1% penicillin-streptomycin at 37 °C in a humidified incubator with 5% CO2. Cells at passage 12–15 were used for all cellular experiments. To generate macrophage cells, THP-1 cells were seeded in 6-cm plates and differentiated with 100 nM phorbol 12-myristate 13-acetate (PMA, HY-18739, MedChemExpress) for 24 h, followed by a 36-h resting period in PMA-free medium. For transcriptome profiling, cells were assigned to four experimental groups: untreated control (CON), AZD7648 (HY-111783, MedChemExpress) alone (AZD), CT DNA (HY-109517, MedChemExpress) alone (DNA), and AZD7648 pretreatment followed by CT DNA stimulation (AZD + DNA). In the AZD group, cells were treated with 3 μM AZD7648 for 7 h. In the DNA group, cells were transfected with 8 μg CT DNA using 12 μL Lipofectamine 3000 (L3000015, Thermo Fisher Scientific) for 6 h. In the AZD + DNA group, cells were pretreated with 3 μM AZD7648 for 1 h and then transfected with 8 μg CT DNA using 12 μL Lipofectamine 3000 for 6 h. Each group contained three biological replicates, resulting in a total of 12 samples. After treatment, cells were lysed in TRIzol reagent (15596026, Invitrogen), snap-frozen in liquid nitrogen, and submitted to Sangon Biotech (Shanghai, China) for RNA extraction, library preparation, and sequencing. The bioinformatics analysis pipeline was independently completed by the authors of this study.

### 4.2 RNA-seq and data quality control analyses

Total RNA was extracted using the Total RNA Extractor (TRIzol) kit (B511311, Sangon, China) according to the manufacturer’s instructions. Genomic DNA contamination was removed by RNase-free DNase I treatment. Sequencing libraries were prepared using the VAHTS mRNA-seq V2 Library Prep Kit for Illumina according to the manufacturer’s protocol. Briefly, mRNA was enriched using poly-T oligo-attached magnetic beads and fragmented under elevated temperature in fragmentation buffer. First-strand cDNA was synthesized using random hexamer primers and M-MuLV reverse transcriptase, followed by second-strand cDNA synthesis using DNA polymerase I and RNase H. After end repair and adenylation of 3′ ends, sequencing adapters were ligated to the cDNA fragments. Libraries with insert sizes of approximately 150–200 bp were purified using the AMPure XP system (Beckman Coulter, Beverly, USA). Adapter-ligated products were treated with USER enzyme (NEB, USA), PCR-amplified using Phusion High-Fidelity DNA polymerase, and purified again using the AMPure XP system. Final library quality was assessed on an Agilent Bioanalyzer 2100 system. Qualified libraries were quantified, pooled, and sequenced on the Illumina NovaSeq platform with paired-end reads.

Raw sequencing data were evaluated using FastQC (v0.11.2). Adapter sequences and low-quality reads were removed using Trimmomatic (v0.36). Filtering steps included removal of adapter-contaminated reads, trimming of low-quality bases from both read ends (Q < 20), sliding-window trimming with a window size of 5 bp, and removal of reads shorter than 35 nt together with their paired reads. The resulting clean reads were used for downstream analyses. Clean reads were aligned to the human reference genome using HISAT2 (v2.0) with default parameters. Alignment statistics were summarized with RSeQC (v2.6.1). Read distribution uniformity and genomic feature assignment were assessed using Qualimap (v2.2.1), and gene coverage statistics were generated using BEDTools (v2.26.0).

### 4.3 Western blot

THP-1 cells grown in 6-cm plates were differentiated with 100 nM PMA for 24 h, followed by a 36-h resting period in PMA-free medium. Then cells were pretreated with 3 μM AZD7648 for 1 h and then transfected with 8 μg CT DNA using 12 μL Lipofectamine 3000 for 6 h. After treatment, cells were collected and lysed in RIPA buffer (50 mM Tris-HCL, 150 mM NaCl, 1% NP-40, 1% sodium deoxycholate, 0.1% SDS, 0.5 mM PMSF, 2 mM Na_3_VO_4_ and proteinase inhibitors). Lysates were sonicated using a Bioruptor sonicator (Diagenode) and clarified by centrifugation at 12,000 × g for 10 min. Protein samples were separated by SDS-PAGE and subjected to immunoblotting with antibodies against γH2AX (05–636, Millipore), H2AX (7631, Cell Signaling Technology), and GAPDH (60004-1-Ig, Proteintech Group).

## Limitations

This dataset is derived from a single cell type (THP-1-derived macrophages) and does not capture transcriptional responses in primary macrophages or other immune cell subsets. In addition, RNA-seq was performed at a single time point (6 h post‑transfection). Therefore, temporal dynamics of gene expression are not represented in this dataset.

## Ethics Statement

The authors have read and follow the ethical requirements for publication in Data in Brief and confirm that the current work does not involve human subjects, animal experiments, or any data collected from social media platforms.

## Ethics Declaration

The author(s) declare(s) that the study does not involve humans nor animal subjects.

## CRediT Author Statement

**Qiang Li**: Sampling, Methodology, Investigation, Validation, Writing – Original Draft; **Pei-Wen Zhan**: Sampling, Formal analysis; **Xian Hong**: Formal analysis, Data curation; **Yong-Pei Li**: Software, Formal analysis; **Xin-Yue Pei**: Software, Data curation, Writing – Review & Editing; **Xiao-Hui Du**: Formal analysis; **Ming Sui**: Investigation; **Tao Wang**: Conceptualization, Project administration, Funding acquisition, Writing – Original Draft, Writing – Review & Editing, Supervision.

## Data Availability

Mendeley DataSupplementary Fig. 2 Original images of western blotting showing (Original data)
SRADataset of RNA-seq profiles from THP-1-derived macrophages pretreated with AZD7648 and stimulated with CT DNA (Original data)

## Appendix Supplementary materials

Image, application 1

Image, application 2

Image, application 3

Image, application 4
