# Supplementary material for: Dataset of RNA-seq profiles from THP-1-derived macrophages pretreated with AZD7648 and stimulated with CT DNA
Source: Data Brief. 2026 Aug 3;68:113124. doi: 10.1016/j.dib.2026.113124 (PMC13486372; doi:10.1016/j.dib.2026.113124)
Supplement: Supplementary file 1 [file mmc1.zip › Figure1B_GSEA_Supporting_Data/Figure1B_CYTOKINE_CYTOKINE_RECEPTOR_INTERACTION.pdf]

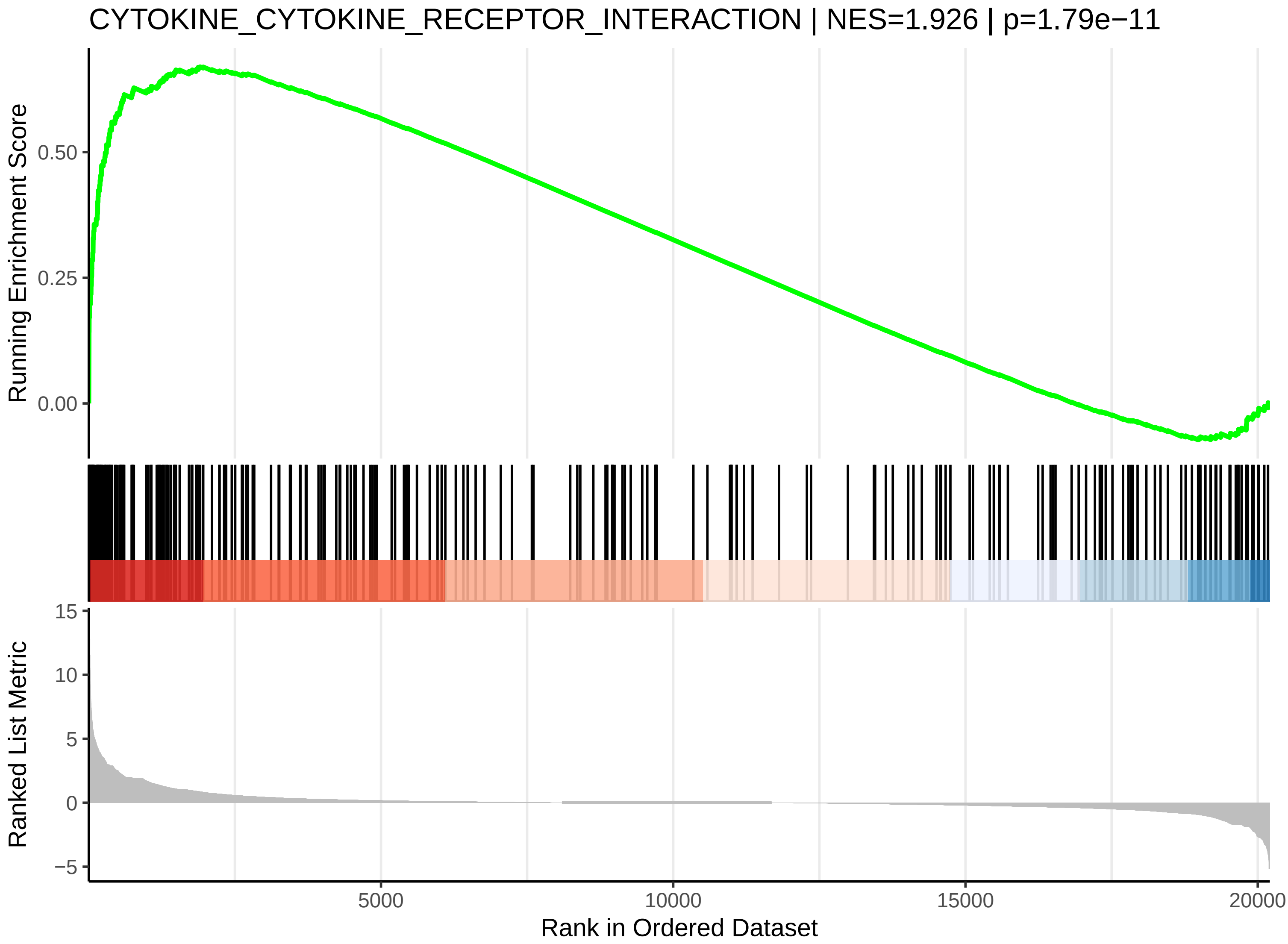
CYTOKINE_CYTOKINE_RECEPTOR_INTERACTION | NES=1.926 | p=1.79e−11
Running Enrichment Score
0.50
0.25
0.00
Ranked List Metric
15
10
5
0
−5
5000
10000
15000
20000
Rank in Ordered Dataset
